# Supplementary material for: Implications of differential size-scaling of cell-cycle regulators on cell size homeostasis
Source: PLoS Comput Biol. 2023 Jul 28;19(7):e1011336. doi: 10.1371/journal.pcbi.1011336 (PMC10411824; doi:10.1371/journal.pcbi.1011336)
Supplement: S1 Appendix — (PDF) [file pcbi.1011336.s001.pdf]

Supplementary Information: Implications of differential size-scaling of cell-cycle regulators on cell size homeostasis

Xiangrui Ji[1], Jie Lin[2,3*]

July 16, 2023

1 Yuanpei College, Peking University, Beijing, China

2 Center for Quantitative Biology, Academy for Advanced Interdisciplinary Studies, Peking University, Beijing, China

3 Peking-Tsinghua Center for Life Sciences, Academy for Advanced Interdisciplinary Studies, Peking University, Beijing, China

* linjie@pku.edu.cn

1 Detailed analysis of the drop in $c_{\rm act}/c_{\rm inh}$ right after division

Because the cell-cycle regulators are degradable, their numbers change much faster than F_n and F_r. Therefore, $F_n(t) \approx F_n(0)$ and $F_r(t) \approx F_r(0)$ right after cell division. For the activator ($i = 3$) and the inhibitor ($i = 4$),

$$\frac{\mathrm{d}m_i}{\mathrm{d}t} = \Gamma_n g_i \frac{F_n(0)c_n}{F_n(0)c_n + K_{n,i}} - \frac{m_i}{\tau_m}, \tag{S1}$$

$$\frac{\mathrm{d}p_i}{\mathrm{d}t} = \Gamma_r m_i \frac{F_r(0)c_r}{F_r(0)c_r + K_r} - \frac{p_i}{\tau_p}. \tag{S2}$$

The solution is

$$p_i(t) = p_i(0)\mathrm{e}^{-\frac{t}{\tau_p}} + \frac{B\tau_m\tau_p}{\tau_p - \tau_m}\left(m_i(0)(\mathrm{e}^{-\frac{t}{\tau_p}} - \mathrm{e}^{-\frac{t}{\tau_m}}) + A_i(\tau_m \mathrm{e}^{-\frac{t}{\tau_m}} - \tau_p \mathrm{e}^{-\frac{t}{\tau_p}} + \tau_p - \tau_m)\right), \tag{S3}$$

where

$$A_i = \Gamma_n g_i \frac{F_n(0)c_n}{F_n(0)c_n + K_{n,i}}, \quad B = \Gamma_r \frac{F_r(0)c_r}{F_r(0)c_r + K_r}. \tag{S4}$$

From Eq S3 and $K_{n,\rm act} > K_{n,\rm inh}$, we get

$$\frac{\mathrm{d}}{\mathrm{d}t}\left(\frac{p_{\rm act}}{p_{\rm inh}}\right) = (A_{\rm act} - A_{\rm inh})C(t) < 0, \tag{S5}$$

where

$$C(t) = \frac{B\tau_m\left(p_i(0)(\tau_m - \tau_p)(\mathrm{e}^{\frac{t}{\tau_m}} - 1) + B\tau_p m_i(0)(\tau_p(\mathrm{e}^{\frac{t}{\tau_p}} - 1) - \tau_m(\mathrm{e}^{\frac{t}{\tau_m}} - 1))\right)}{(\tau_m - \tau_p)\mathrm{e}^{\frac{t}{\tau_m} + \frac{t}{\tau_p}} p_{\rm inh}^2} > 0. \tag{S6}$$

Therefore, $c_{\rm act}/c_{\rm inh}$ decreases right after cell birth. Additionally, from Eq S3, we find that in the limit of very small τ_m and τ_p, the decrease of $c_{\rm act}/c_{\rm inh}$ is very sharp. A similar analysis applies to the modified model with gene replication in Section 4 of S1 Appendix.

2 The stability analysis of the cell cycle

In this section, we prove the stability of the cell cycle in the deterministic model. When the ratio of the activator to the inhibitor reaches the threshold value, the system is on the $2N-1$ dimensional hyperplane $\theta = \frac{p_{\text{act}}}{p_{\text{inh}}}$ in the phase space, where N is the total number of genes. Right after division, it is still on this hyperplane because all mRNAs and proteins halve. Without loss of generality, we use the quantities right after division to describe the system's state. We denote $\boldsymbol{x}_k = (m_{1,k}, ..., m_{N,k}, n_k, p_{\text{inh},k}, r_k, p_{5,k}, ..., p_{N,k})^T$ as the state vector of the system when it reaches this hyperplane for the k-th time. Note that we exclude $p_{\text{act},k}$ from the state vector for it is identically equal to $\theta p_{\text{inh},k}$ on the hyperplane. The Poincaré map [1] P satisfies $\boldsymbol{x}_k = P(\boldsymbol{x}_{k-1})$. In the following we prove that the Jacobian matrix of P only has eigenvalues with absolute values smaller than 1.

For nondegradable proteins, the solution to Eq 8 is

$$p_i(t) = p_i(0) + \Gamma_{n,i} g_i \tau_m \Gamma_r \int_0^t \frac{F_n(s)c_n}{F_n(s)c_n + K_n} \frac{F_r(s)c_r}{F_r(s)c_r + K_r} \mathrm{d}s, \tag{S7}$$

In particular,

$$n(t) = n(0) + \Gamma_{n,n} g_n \tau_m \Gamma_r \int_0^t \frac{F_n(s)c_n}{F_n(s)c_n + K_n} \frac{F_r(s)c_r}{F_r(s)c_r + K_r} \mathrm{d}s, \tag{S8}$$

Therefore,

$$p_i(t) = p_i(0) + \frac{\Gamma_{n,i} g_i}{\Gamma_{n,n} g_n}(n(t) - n(0)). \tag{S9}$$

According to Eqs 20,21,

$$m_{i,k} = \Gamma_{n,i} g_i \tau_m \frac{\tilde{K}}{2((1-\theta)K_n + \tilde{K})}, \quad k \in \mathbb{N}_+, \tag{S10}$$

$$n_k = \frac{n_c c_n (1-\theta)\tilde{K}}{2((1-\theta)c_n - \tilde{K})((1-\theta)K_n + \tilde{K})}, \quad k \in \mathbb{N}_+. \tag{S11}$$

For proteins except RNAPs and cell-cycle regulators, from Eq S9,

$$2p_{i,k} = p_{i,k-1} + \frac{\Gamma_{n,i} g_i}{\Gamma_{n,n} g_n}(2n_k - n_{k-1}) = p_{i,k-1} + \frac{\Gamma_{n,i} g_i}{\Gamma_{n,n} g_n} \frac{n_c c_n (1-\theta)\tilde{K}}{2((1-\theta)c_n - \tilde{K})((1-\theta)K_n + \tilde{K})}, \quad k \in \mathbb{N}_+. \tag{S12}$$

From Eq 16,

$$2p_{\text{inh},k} = \Gamma_n g_{\text{inh}} \tau_m \Gamma_r \tau_p \frac{F_{n,d} c_n}{F_{n,d} c_n + K_{n,\text{inh}}} \frac{F_{r,d} c_r}{F_{r,d} c_r + K_r}, \tag{S13}$$

where the fraction of free RNAPs at cell division $F_{n,d}$ is a constant (Eq 17), while the fraction of free ribosomes at cell division $F_{r,d}$ depends on r_{k-1} (Eq 9 and Eq S12 about r_k). Therefore, from Eqs S10-S13, $\frac{\partial \boldsymbol{x}_k}{\partial \boldsymbol{x}_{k-1}}$ is an upper-triangular matrix with diagonal entries $\underbrace{0, ..., 0}_{N+2}, \underbrace{\frac{1}{2}, ..., \frac{1}{2}}_{N-3}$. The eigenvalues of the Jacobian matrix are precisely these diagonal entries, and their absolute values are all small than 1. Therefore, the periodic orbit is stable against infinitesimal perturbation.

3 Imperfect sizer

To accommodate the fact that cells display imperfect sizers [2, 3, 4, 5, 6, 7, 8], we assume that cell division is a stochastic process with its probability increasing with the activator-to-inhibitor ratio. This idea is similar

to some previous work [3, 9, 10, 11, 12]. Specifically, we first let the cell grow for a short period in order to avoid the transient effect right after division and prevent immediate cell division without significant growth, similar to the simulations in [12]. After this period, the probability for a cell with the activator-to-inhibitor ratio in the infinitesimal interval $[\theta, \theta + \mathrm{d}\theta)$ to divide is $k(\theta)\mathrm{d}\theta$. The probability distribution for the ratio θ at division is

$$p(\theta) = k(\theta)\mathrm{e}^{-\int_{\theta_b}^{\theta} k(\theta^{'})\mathrm{d}\theta^{'}}, \tag{S14}$$

where θ_b is the activator-to-inhibitor ratio right after the short period. To ensure $\int_{\theta_b}^{\theta_2} p(\theta)\mathrm{d}\theta = 1$, where θ_2 is the larger critical threshold value in the main text, $k(\theta)$ must satisfy $\lim_{\theta \to \theta_2} k(\theta) = +\infty$.

The cell volume V can be expressed by an increasing function $V(\theta)$, because the ratio θ between the regulators undergoing size-dependent expression is a measurement for cell volume. The expectation for the division size V_d is then

$$\langle V_d \rangle = \int_{\theta_b}^{\theta_2} V(\theta)p(\theta)\mathrm{d}\theta = \int_{\theta_b}^{\theta_2} V(\theta)k(\theta)\mathrm{e}^{-\int_{\theta_b}^{\theta} k(\theta^{'})\mathrm{d}\theta^{'}}\mathrm{d}\theta. \tag{S15}$$

We consider a cell born with a larger size. The expectation for its division size $V_d^{'}$ is then

$$\langle V_d^{'} \rangle = \int_{\theta_b^{'}}^{\theta_2} V(\theta)k(\theta)\mathrm{e}^{-\int_{\theta_b^{'}}^{\theta} k(\theta^{'})\mathrm{d}\theta^{'}}\mathrm{d}\theta, \tag{S16}$$

where $\theta_b^{'} > \theta_b$. If $k(\theta)$ becomes nonzero before θ reaches $\theta_b^{'}$,

$$\begin{aligned}
\langle V_d^{'} \rangle - \langle V_d \rangle &= \int_{\theta_b^{'}}^{\theta_2} V(\theta)k(\theta)\left(\mathrm{e}^{-\int_{\theta_b^{'}}^{\theta} k(\theta^{'})\mathrm{d}\theta^{'}} - \mathrm{e}^{-\int_{\theta_b}^{\theta} k(\theta^{'})\mathrm{d}\theta^{'}}\right)\mathrm{d}\theta - \int_{\theta_b}^{\theta_b^{'}} V(\theta)k(\theta)\mathrm{e}^{-\int_{\theta_b}^{\theta} k(\theta^{'})\mathrm{d}\theta^{'}}\mathrm{d}\theta \\
&> V(\theta_b^{'})\int_{\theta_b^{'}}^{\theta_2} k(\theta)\left(\mathrm{e}^{-\int_{\theta_b^{'}}^{\theta} k(\theta^{'})\mathrm{d}\theta^{'}} - \mathrm{e}^{-\int_{\theta_b}^{\theta} k(\theta^{'})\mathrm{d}\theta^{'}}\right)\mathrm{d}\theta - V(\theta_b^{'})\int_{\theta_b}^{\theta_b^{'}} k(\theta)\mathrm{e}^{-\int_{\theta_b}^{\theta} k(\theta^{'})\mathrm{d}\theta^{'}}\mathrm{d}\theta \\
&= V(\theta_b^{'})\left(1 - \int_{\theta_b^{'}}^{\theta_2} k(\theta)\mathrm{e}^{-\int_{\theta_b}^{\theta} k(\theta^{'})\mathrm{d}\theta^{'}}\mathrm{d}\theta\right) - V(\theta_b^{'})\int_{\theta_b}^{\theta_b^{'}} k(\theta)\mathrm{e}^{-\int_{\theta_b}^{\theta} k(\theta^{'})\mathrm{d}\theta^{'}}\mathrm{d}\theta = 0,
\end{aligned} \tag{S17}$$

which means $\langle V_d \rangle$ increases with θ_b. Therefore, $\langle V_d \rangle$ increases with the birth volume, which is the imperfect sizer. Intuitively, the cell with a smaller birth size is more likely to divide at a smaller size on average because it has a finite chance to divide before it reaches the birth size of the larger cell (S9A Fig).

As a simple example, we assume

$$k(\theta) = \begin{cases} C_1\left(\frac{\theta - \theta_1}{\theta_2 - \theta}\right)^{C_2}, & \theta_1 < \theta < \theta_2 \\ 0, & \text{others} \end{cases}. \tag{S18}$$

where the constant $C_1, C_2 > 0$ and θ_1 is the smaller critical threshold value in the main text. We numerically generate imperfect sizers with the slopes similar to experiments (S9B-S9G Fig). We note that the model with stochastic cell division may lead to a division size close to the birth size. It cannot generate control strategies far from the sizer, such as a perfect adder. This is because it does not effectively measure the size increment. Nevertheless, it can be considered a minimal model for an imperfect sizer. The division probability density k can also depend on other variables such as the time spent in the cell cycle, which may render a weaker size control such as adder [13]. We would like to clarify that the phenomenological adder (or near-adder) over the entire cell cycle observed in eukaryotic cells [7, 14] can emerge from the combination of independent regulations at different cell-cycle stages [3, 15, 16].

Finally, we want to mention that for any distribution $p(\theta)$, there exists a corresponding distribution $k(\theta)$. Integrating over both sides of Eq S14, we get

$$\int_{\theta_b}^{x} p(\theta)\mathrm{d}\theta = 1 - \mathrm{e}^{-\int_{\theta_b}^{x} k(\theta')\mathrm{d}\theta'}, \tag{S19}$$

$$\int_{\theta_b}^{x} k(\theta')\mathrm{d}\theta' = -\ln\left(1 - \int_{\theta_b}^{x} p(\theta)\mathrm{d}\theta\right). \tag{S20}$$

Taking the derivative on both sides of Eq S20, we can determine $k(\theta)$ from $p(\theta)$,

$$k(\theta) = \frac{p(\theta)}{1 - \int_{\theta_b}^{\theta} p(\theta')\mathrm{d}\theta'}. \tag{S21}$$

4 The modified model with gene replication

In this section, we assume that when the ratio of the activator to the inhibitor $c_{\mathrm{act}}/c_{\mathrm{inh}}$ rises to the threshold θ, the cell replicates its genes instantly and divides after a constant time T_M. Like the simplified model in the main text, here the cell can also reach a periodic steady state (S6A-S6C Fig). The difference is that here when genes replicate, the free RNAP fraction F_n drops abruptly (S6D Fig), causing the reduction of $c_{\mathrm{act}}/c_{\mathrm{inh}}$.

We can easily obtain the expression of V_b after some modification to the corresponding deviation in Methods. We use subscript S to denote the values at gene replication. Substituting Eqs 15-16 into

$$\theta = \frac{p_{\mathrm{act},S}}{p_{\mathrm{inh},S}}, \tag{S22}$$

we obtain

$$F_{n,S} = \frac{\theta K_{n,\mathrm{act}} - K_{n,\mathrm{inh}}}{(1-\theta)c_n}. \tag{S23}$$

Substituting Eq S23 into Eq 18, we get the critical size [17] to enter S phase

$$V_S = \frac{a}{c_n} n_S = \frac{a n_c (1-\theta)\tilde{K}}{((1-\theta)c_n - \tilde{K})((1-\theta)K_n + \tilde{K})}, \tag{S24}$$

$$V_b = \frac{1}{2} V_S \mathrm{e}^{\mu T_M} = \frac{\mathrm{e}^{\mu T_M} a n_c (1-\theta)\tilde{K}}{2((1-\theta)c_n - \tilde{K})((1-\theta)K_n + \tilde{K})}. \tag{S25}$$

When $T_M = 0$, Eq S25 becomes the same as Eq 2 in the main text. Noticing the constrain of $0 < F_{n,S} < 1$ in Eq S23, we get the range for θ, which is the same as Eq 4.

We estimate the growth rate μ in Eq S25 as follows. Substituting Eq 13 about r and Eq 18 into Eq 33, we get

$$\frac{F_r c_r}{F_r c_r + K_r} \approx \frac{n_c \Gamma_{n,r} g_r (1 - F_r)}{\Gamma_{n,n} g_n \tau_m (1 - F_n) \sum_i \Gamma_{n,i} g_i \left(1 + \frac{\Gamma_r L_i}{v_r}\right)}. \tag{S26}$$

When $F_n \ll 1$, Eq S26 becomes

$$\frac{F_r c_r}{F_r c_r + K_r} \approx \frac{n_c \Gamma_{n,r} g_r (1 - F_r)}{\Gamma_{n,n} g_n \tau_m \sum_i \Gamma_{n,i} g_i \left(1 + \frac{\Gamma_r L_i}{v_r}\right)}, \tag{S27}$$

from which we can determine F_r. Then, from Eq 34, we obtain the growth rate μ. The constant μ means that the cell grows exponentially. This scenario is called Phase 1 of gene expression [18]. Although a large cell deviates from Phase 1, its growth rate changes relatively mildly, so the μ calculated above is still a good approximation. Comparison between the predictions with simulations shows good agreement (S6E Fig).

5 The case of degradable activator and nondegradable inhibitor

Although there are many short-lived cell-cycle regulators, such as the activator Cln3 [19, 20] and the inhibitor Sic1 [21, 22, 23] in budding yeast, the inhibitor Whi5 degrades slowly [24, 25]. In mammalian cells, the cell-cycle inhibitor Rb is also very stable [26, 27, 28]. Therefore, the case of degradable activator but nondegradable inhibitor is also worth discussion. We set the transcription initiation rate of the inhibitor to be Γ_n/α ($\alpha > 1$) so that the level of activators and inhibitors are more comparable.

5.1 Derivation of the cell size at birth and the two critical threshold values

Combining Eqs 8,11, we get

$$\frac{\mathrm{d}}{\mathrm{d}t}\left(\frac{p_{\mathrm{inh}}}{n}\right) = \frac{\Gamma_{n,n} g_n \tau_m \Gamma_r}{n} \frac{F_n c_n}{F_n c_n + K_n} \frac{F_r c_r}{F_r c_r + K_r} \left(\frac{\Gamma_n g_{\mathrm{inh}}}{\alpha \Gamma_{n,n} g_n} \frac{F_n c_n + K_n}{F_n c_n + K_{n,\mathrm{inh}}} - \frac{p_{\mathrm{inh}}}{n}\right). \tag{S28}$$

We use quasi-steady-state approximation for $\frac{p_{\mathrm{inh}}}{n}$ at division: $\frac{\mathrm{d}}{\mathrm{d}t}\left(\frac{p_{\mathrm{inh}}}{n}\right) = 0$. Therefore,

$$p_{\mathrm{inh},d} = \frac{\Gamma_n g_{\mathrm{inh}}}{\alpha \Gamma_{n,n} g_n} \frac{F_{n,d} c_n + K_n}{F_{n,d} c_n + K_{n,\mathrm{inh}}} n_d. \tag{S29}$$

In the simple case of $g_{\mathrm{act}} = g_{\mathrm{inh}} = 1$, substituting Eqs 15,S29 into Eq 1, and using Eq 18, we get

$$\frac{\theta}{\alpha} = \frac{\Gamma_{n,n} g_n \tau_m \Gamma_r \tau_p}{n_c} \frac{(1 - F_{n,d})(F_{n,d} c_n + K_{n,\mathrm{inh}})}{F_{n,d} c_n + K_{n,\mathrm{act}}} \frac{F_{r,d} c_r}{F_{r,d} c_r + K_r}. \tag{S30}$$

From Eqs S26,S30, we can numerically solve $F_{n,d}$, and eventually get n_b and V_b (S7A Fig, Prediction 1).

Experimentally, cell growth in an unperturbed cell cycle is typically nearly exponential [29, 30, 31], so we are especially concerned about the cell in Phase 1. According to [32], for the cell in Phase 1,

$$p_{\mathrm{act}}(t) = \frac{\Gamma_n K_n n_c}{\Gamma_{n,n}} \frac{\mu \tau_p n(0) \mathrm{e}^{\mu t}}{K_{n,\mathrm{act}} n_c - (K_{n,\mathrm{act}} - K_n) n(0) \mathrm{e}^{\mu t}}, \tag{S31}$$

$$p_{\mathrm{inh}}(t) = p_{\mathrm{inh}}(0) + \frac{\Gamma_n K_n n_c}{\alpha \Gamma_{n,n}} \frac{1}{K_n - K_{n,\mathrm{inh}}} \ln \frac{K_{n,\mathrm{inh}} n_c - (K_{n,\mathrm{inh}} - K_n) n(0) \mathrm{e}^{\mu t}}{K_{n,\mathrm{inh}} n_c - (K_{n,\mathrm{inh}} - K_n) n(0)}, \tag{S32}$$

where μ is estimated as Section 4 of S1 Appendix. Using $p_{\mathrm{inh},d} = 2 p_{\mathrm{inh},b}$, we get

$$p_{\mathrm{inh},d} = \frac{2 \Gamma_n K_n n_c}{\alpha \Gamma_{n,n}} \frac{1}{K_n - K_{n,\mathrm{inh}}} \ln \frac{K_{n,\mathrm{inh}} n_c - 2(K_{n,\mathrm{inh}} - K_n) n_b}{K_{n,\mathrm{inh}} n_c - (K_{n,\mathrm{inh}} - K_n) n_b}, \tag{S33}$$

Substituting Eqs S31,S33 into Eq 1, we obtain

$$\frac{\theta}{\alpha} = \frac{\frac{\mu \tau_p n_b}{K_{n,\mathrm{act}} n_c - 2(K_{n,\mathrm{act}} - K_n) n_b}}{\frac{1}{K_n - K_{n,\mathrm{inh}}} \ln \frac{K_{n,\mathrm{inh}} n_c - 2(K_{n,\mathrm{inh}} - K_n) n_b}{K_{n,\mathrm{inh}} n_c - (K_{n,\mathrm{inh}} - K_n) n_b}}. \tag{S34}$$

Eq S34 can implicitly determine n_b and V_b. To get an approximate explicit solution for n_b, we use Taylor expansion

$$\begin{aligned} \frac{1}{K_n - K_{n,\mathrm{inh}}} \ln \frac{K_{n,\mathrm{inh}} n_c - 2(K_{n,\mathrm{inh}} - K_n) n_b}{K_{n,\mathrm{inh}} n_c - (K_{n,\mathrm{inh}} - K_n) n_b} &= \frac{1}{K_n - K_{n,\mathrm{inh}}} \ln \left(1 + \frac{(K_n - K_{n,\mathrm{inh}}) n_b}{K_{n,\mathrm{inh}} n_c - (K_{n,\mathrm{inh}} - K_n) n_b}\right) \\ &\approx \frac{n_b}{K_{n,\mathrm{inh}} n_c - (K_{n,\mathrm{inh}} - K_n) n_b}, \end{aligned} \tag{S35}$$

so we get

$$n_b \approx \frac{n_c (\theta K_{n,\mathrm{act}}/\alpha - \mu \tau_p K_{n,\mathrm{inh}})}{2\theta (K_{n,\mathrm{act}} - K_n)/\alpha + \mu \tau_p (K_n - K_{n,\mathrm{inh}})}, \tag{S36}$$

Finally, we obtain V_b (S7A Fig, Prediction 2), which fits well with simulations (S7A Fig) for relatively small V_b. The deviation for large V_b is reasonable, because Eqs S31-S32 are no longer accurate after the cell leaves Phase 1. Letting $n_b \to 0$ in Eq S34 or Eq S36, we find that one of the critical thresholds is $\theta_1 = \frac{\alpha\mu\tau_p K_{n,\mathrm{inh}}}{K_{n,\mathrm{act}}}$. It's hard to analytically calculate the other critical point θ_2, but S7B Fig provides an illustration. When the cell is excessively large, genes and mRNAs get saturated, so the number of the degradable activator becomes constant, and the number of the nondegradable inhibitor is proportional to cell volume. Therefore, $c_{\mathrm{act}}/c_{\mathrm{inh}}$ approaches to zero, and the cell cannot divide any more. The phenomenon that large cell volume prevents cell division is consistent with experiments [33, 34, 35].

5.2 The correlations between V_b and V_d

As in the main text, we consider the noise in the threshold value. Given a θ, from Eqs 18,S26,S30 we can determine the number of RNAPs at cell division n_d and then the cell size at division V_d approximately. Therefore, V_d in a certain generation nearly only depends on the threshold of that generation and is consequently uncorrelated with V_b. This prediction is close to simulations (S7C-S7E Fig) and experiments [2, 8].

6 Alternative criteria for cell division

In this section, we explore alternative criteria that govern cell-cycle progression. We first assume that the cell divides once the concentration c_i of one activator or inhibitor i reaches a threshold value θ_i. When this event happens,

$$c_i = \frac{p_i}{V} = \frac{c_n p_i}{an} = \theta_i. \tag{S37}$$

The cell volume at birth $V_{b,i} = c_i^{-1}(\theta_i)/2$, where c_i^{-1} denotes the inverse function of $c_i(V)$ (S8 Fig).

If the regulator i is degradable, substituting Eq 15 or Eq 16 into Eq S37, and using Eq 18, we get

$$\tilde{\theta}_i = \frac{\Gamma_{n,n} g_n \tau_m \Gamma_r \tau_p}{n_c} \frac{(1 - F_{n,d})(F_{n,d} c_n + K_n)}{F_{n,d} c_n + K_{n,i}} \frac{F_{r,d} c_r}{F_{r,d} c_r + K_r}. \tag{S38}$$

where $\tilde{\theta}_i \equiv \frac{a\Gamma_{n,n} g_n}{c_n \Gamma_{n,i} g_i} \theta_i$. From Eqs S26,S38, we can numerically solve $F_{n,d}$, and eventually get V_b. The derivation here is similar to Section 5 of S1 Appendix, and we can also obtain the critical threshold $\tilde{\theta}_{i,1} = \frac{\mu\tau_p K_n}{K_{n,i}}$ (S8A and S8B Fig).

If the regulator i is nondegradable, substituting Eq S29 into Eq S37, we get

$$F_{n,d} = \frac{\tilde{\theta}_i K_{n,i} - K_n}{(1 - \tilde{\theta}_i) c_n}. \tag{S39}$$

Similar to the derivation in Methods, we obtain the cell volume at birth (S8C Fig)

$$V_{b,i} = \frac{a n_c (1 - \tilde{\theta}_i)(\tilde{\theta}_i K_{n,i} - K_n)}{2((1 - \tilde{\theta}_i) c_n - (\tilde{\theta}_i K_{n,i} - K_n))((1 - \tilde{\theta}_i) K_n + (\tilde{\theta}_i K_{n,i} - K_n))}, \tag{S40}$$

where

$$\frac{K_n}{K_{n,i}} < \tilde{\theta}_i < \frac{c_n + K_n}{c_n + K_{n,i}}. \tag{S41}$$

We then consider the logic gates for multiple regulators motivated by the AND gate logic observed in *E. coli* [36, 37]. For the AND gate, i.e., the cell divides after each c_i reaches its own threshold θ_i, the birth volume $V_{b,\text{AND}} = \max\{V_{b,i}\}$ (S8D Fig). For the OR gate, i.e., the cell divides once one of the c_i reaches its threshold, the birth volume $V_{b,\text{OR}} = \min\{V_{b,i}\}$. Similar methods can be applied to the combination of AND and OR gates. We note that the combination of logic gates cannot explain the phenotype of some mutants. For example, *cln3*Δ*bck2*Δ for budding yeast is lethal, while *cln3*Δ*bck2*Δ*whi5*Δ is viable [38], which we think is beyond a combination of logic gates.

7 Asymmetric division

In this section, we consider asymmetric division to better apply our model to asymmetrically dividing organisms such as budding yeast [39]. The proportion of the cell volume the daughter cell inherits is $\gamma < 1/2$.

For the case of degradable activator and inhibitor, using $n_b = \gamma n_d$ and Eq 21, we easily get the cell volume at birth for the daughter cell

$$V_b = \frac{\gamma a n_c (1-\theta)\tilde{K}}{((1-\theta)c_n - \tilde{K})((1-\theta)K_n + \tilde{K})}. \tag{S42}$$

The two critical thresholds remain the same (S11A Fig).

We also consider a modified model in which the inhibitor is nondegradable and some inhibitor molecules are bound to chromatins at cell division as Whi5 in budding yeast [24, 25, 40]. Therefore, the proportion η of the inhibitor molecules that the daughter cell inherits satisfies $\gamma < \eta < 1/2$. Similar to derivations in Section 5 of S1 Appendix, using $n_b = \gamma n_d$ and $p_{\text{inh},b} = \eta p_{\text{inh},d}$ instead, we obtain

$$n_b \approx \frac{n_c\left(\frac{(1-\gamma)\theta}{(1-\eta)\alpha}K_{n,\text{act}} - \mu\tau_p K_{n,\text{inh}}\right)}{\frac{1}{\gamma}\frac{(1-\gamma)\theta}{(1-\eta)\alpha}(K_{n,\text{act}} - K_n) + \mu\tau_p(K_n - K_{n,\text{inh}})}. \tag{S43}$$

When $\gamma = \eta = 1/2$, Eq S43 becomes Eq S36. The smaller critical threshold $\theta_1 = \frac{(1-\eta)\alpha\mu\tau_p K_{n,\text{inh}}}{(1-\gamma)K_{n,\text{act}}}$. Simulations confirm our predictions (S11B Fig).

In the following, we assume that the cell replicates its genes when the activator-to-inhibitor ratio rises to a threshold and then divides after a constant time T_M. The cell can reach a periodic steady state (S11C-S11E Fig). The difference is that here both when genes replicate and when the cell divides, the free RNAP fraction F_n drops abruptly (S11F Fig), causing the reduction of $c_{\text{act}}/c_{\text{inh}}$. Due to the chromatin-based partitioning of the inhibitor, c_{inh} increases abruptly at cell birth (S11C Fig). Interestingly, the mRNA number and protein synthesis rate of the inhibitor increase after G1/S transition (S11G Fig), which is similar to the behavior of Whi5 synthesis in budding yeast [24, 25]. We remark that our model predicts that the *WHI5* mRNA number and Whi5 synthesis rate in S/G2/M are approximately twice as much as those in G1 because of the doubled *WHI5* gene copy number and the strong affinity of the *WHI5* promoter with RNAP. This prediction falls between the two opposite views ([41] vs. [42, 43]). Specific regulation on Whi5 synthesis seems to exist in different cell-cycle stages. We acknowledge that we cannot quantitatively compare the oscillation amplitude in our model with that of Whi5, given that the behavior and regulation of Whi5 synthesis remains an open question.

In summary, asymmetric division does not lead to qualitative difference in our results, and our methodology can be applied to this case with minor modifications.

References

1. Strogatz SH. Nonlinear Dynamics and Chaos: With Applications to Physics, Biology, Chemistry, and Engineering. Boca Raton: CRC Press; 2018.

2. Turner JJ, Ewald JC, Skotheim JM. Cell size control in yeast. Current Biology. 2012;22(9):R350–R359. doi:10.1016/j.cub.2012.02.041.

3. Chandler-Brown D, Schmoller KM, Winetraub Y, Skotheim JM. The adder phenomenon emerges from independent control of pre- and post-Start phases of the budding yeast cell cycle. Current Biology. 2017;27(18):2774–2783.e3. doi:10.1016/j.cub.2017.08.015.

4. Fantes PA. Control of cell size and cycle time in Schizosaccharomyces pombe. Journal of Cell Science. 1977;24:51–67. doi:10.1242/jcs.24.1.51.

5. Sveiczer A, Novak B, Mitchison JM. The size control of fission yeast revisited. Journal of Cell Science. 1996;109(12):2947–2957. doi:10.1242/jcs.109.12.2947.

6. Wood E, Nurse P. Pom1 and cell size homeostasis in fission yeast. Cell Cycle. 2013;12(19):3228–3236. doi:10.4161/cc.26462.

7. Cadart C, Monnier S, Grilli J, Sáez PJ, Srivastava N, Attia R, et al. Size control in mammalian cells involves modulation of both growth rate and cell cycle duration. Nature Communications. 2018;9(1):3275. doi:10.1038/s41467-018-05393-0.

8. Xie S, Skotheim JM. A G1 sizer coordinates growth and division in the mouse epidermis. Current Biology. 2020;30(5):916–924.e2. doi:10.1016/j.cub.2019.12.062.

9. Lord PG, Wheals AE. Variability in individual cell cycles of Saccharomyces cerevisiae. Journal of Cell Science. 1981;50:361–376. doi:10.1242/jcs.50.1.361.

10. Wheals AE. Size control models of Saccharomyces cerevisiae cell proliferation. Molecular and Cellular Biology. 1982;2(4):361–368. doi:10.1128/MCB.2.4.361.

11. Tyson JJ, Diekmann O. Sloppy size control of the cell division cycle. Journal of Theoretical Biology. 1986;118(4):405–426. doi:10.1016/S0022-5193(86)80162-X.

12. Patterson JO, Rees P, Nurse P. Noisy cell-size-correlated expression of cyclin B drives probabilistic cell-size homeostasis in fission yeast. Current Biology. 2019;29(8):1379–1386.e4. doi:10.1016/j.cub.2019.03.011.

13. Osella M, Nugent E, Lagomarsino MC. Concerted control of Escherichia coli cell division. Proceedings of the National Academy of Sciences of the United States of America. 2014;111(9):3431–3435. doi:10.1073/pnas.1313715111.

14. Soifer I, Robert L, Amir A. Single-cell analysis of growth in budding yeast and bacteria reveals a common size regulation strategy. Current Biology. 2016;26(3):356–361. doi:10.1016/j.cub.2015.11.067.

15. Heldt FS, Lunstone R, Tyson JJ, Novák B. Dilution and titration of cell-cycle regulators may control cell size in budding yeast. PLOS Computational Biology. 2018;14(10):e1006548. doi:10.1371/journal.pcbi.1006548.

16. Xie S, Swaffer M, Skotheim JM. Eukaryotic cell size control and its relation to biosynthesis and senescence. Annual Review of Cell and Developmental Biology. 2022;38:291–319. doi:10.1146/annurev-cellbio-120219-040142.

17. Johnston GC, Pringle JR, Hartwell LH. Coordination of growth with cell division in the yeast Saccharomyces cerevisiae. Experimental Cell Research. 1977;105(1):79–98. doi:10.1016/0014-4827(77)90154-9.

18. Lin J, Amir A. Homeostasis of protein and mRNA concentrations in growing cells. Nature Communications. 2018;9(1):4496. doi:10.1038/s41467-018-06714-z.

19. Tyers M, Tokiwa G, Nash R, Futcher B. The Cln3-Cdc28 kinase complex of S. cerevisiae is regulated by proteolysis and phosphorylation. EMBO Journal. 1992;11(5):1773–1784. doi:10.1002/j.1460-2075.1992.tb05229.x.

20. Cross FR, Blake CM. The yeast Cln3 protein is an unstable activator of Cdc28. Molecular and Cellular Biology. 1993;13(6):3266–3271. doi:10.1128/MCB.13.6.3266.

21. Schwob E, Böhm T, Mendenhall MD, Nasmyth K. The B-type cyclin kinase inhibitor p40^{SIC1} controls the G1 to S transition in S. cerevisiae. Cell. 1994;79(2):233–244. doi:10.1016/0092-8674(94)90193-7.

22. Schneider BL, Yang QH, Futcher AB. Linkage of replication to start by the Cdk inhibitor Sic1. Science. 1996;272(5261):560–562. doi:10.1126/science.272.5261.560.

23. Verma R, Annan RS, Huddleston MJ, Carr SA, Reynard G, Deshaies RJ. Phosphorylation of Sic1p by G1 Cdk required for its degradation and entry into S phase. Science. 1997;278(5337):455–460. doi:10.1126/science.278.5337.455.

24. Schmoller KM, Turner JJ, Kõivomägi M, Skotheim JM. Dilution of the cell cycle inhibitor Whi5 controls budding-yeast cell size. Nature. 2015;526(7572):268–272. doi:10.1038/nature14908.

25. Qu Y, Jiang J, Liu X, Wei P, Yang X, Tang C. Cell cycle inhibitor Whi5 records environmental information to coordinate growth and division in yeast. Cell Reports. 2019;29(4):987–994. doi:10.1016/j.celrep.2019.09.030.

26. Boyer SN, Wazer DE, Band V. E7 protein of human papilloma virus-16 induces degradation of retinoblastoma protein through the ubiquitin-proteasome pathway. Cancer Research. 1996;56(20):4620–4624.

27. Wang Y, Zheng Z, Zhang J, Wang Y, Kong R, Liu J, et al. A novel retinoblastoma protein (RB) E3 ubiquitin ligase (NRBE3) promotes RB degradation and is transcriptionally regulated by E2F1 transcription factor. Journal of Biological Chemistry. 2015;290(47):28200–28213. doi:10.1074/jbc.M115.655597.

28. Zatulovskiy E, Zhang S, Berenson DF, Topacio BR, Skotheim JM. Cell growth dilutes the cell cycle inhibitor Rb to trigger cell division. Science. 2020;369(6502):466–471. doi:10.1126/science.aaz6213.

29. Elliott SG, McLaughlin CS. Rate of macromolecular synthesis through the cell cycle of the yeast Saccharomyces cerevisiae. Proceedings of the National Academy of Sciences of the United States of America. 1978;75(9):4384–4388. doi:10.1073/pnas.75.9.4384.

30. Di Talia S, Skotheim JM, Bean JM, Siggia ED, Cross FR. The effects of molecular noise and size control on variability in the budding yeast cell cycle. Nature. 2007;448(7156):947–951. doi:10.1038/nature06072.

31. Godin M, Delgado FF, Son S, Grover WH, Bryan AK, Tzur A, et al. Using buoyant mass to measure the growth of single cells. Nature Methods. 2010;7(5):387–390. doi:10.1038/nmeth.1452.

32. Wang Q, Lin J. Heterogeneous recruitment abilities to RNA polymerases generate nonlinear scaling of gene expression with cell volume. Nature Communications. 2021;12(1):6852. doi:10.1038/s41467-021-26952-y.

33. Neurohr GE, Terry RL, Lengefeld J, Bonney M, Brittingham GP, Moretto F, et al. Excessive cell growth causes cytoplasm dilution and contributes to senescence. Cell. 2019;176(5):1083–1097.e18. doi:10.1016/j.cell.2019.01.018.

34. Lengefeld J, Cheng CW, Maretich P, Blair M, Hagen H, McReynolds MR, et al. Cell size is a determinant of stem cell potential during aging. Science Advances. 2021;7(46):eabk0271. doi:10.1126/sciadv.abk0271.

35. Lanz MC, Zatulovskiy E, Swaffer MP, Zhang L, Ilerten I, Zhang S, et al. Increasing cell size remodels the proteome and promotes senescence. Molecular Cell. 2022;82(17):3255–3269.e8. doi:10.1016/j.molcel.2022.07.017.

36. Micali G, Grilli J, Osella M, Lagomarsino MC. Concurrent processes set E. coli cell division. Science Advances. 2018;4(11):eaau3324. doi:10.1126/sciadv.aau3324.

37. Colin A, Micali G, Faure L, Lagomarsino MC, van Teeffelen S. Two different cell-cycle processes determine the timing of cell division in Escherichia coli. eLife. 2021;10:e67495. doi:10.7554/eLife.67495.

38. Adames NR, Schuck PL, Chen KC, Murali TM, Tyson JJ, Peccoud J. Experimental testing of a new integrated model of the budding yeast Start transition. Molecular Biology of the Cell. 2015;26(22):3966–3984. doi:10.1091/mbc.E15-06-0358.

39. Hartwell LH, Unger MW. Unequal division in Saccharomyces cerevisiae and its implications for the control of cell division. Journal of Cell Biology. 1977;75(2):422–435. doi:10.1083/jcb.75.2.422.

40. Swaffer MP, Kim J, Chandler-Brown D, Langhinrichs M, Marinov GK, Greenleaf WJ, et al. Transcriptional and chromatin-based partitioning mechanisms uncouple protein scaling from cell size. Molecular Cell. 2021;81(23):4861–4875.e7. doi:10.1016/j.molcel.2021.10.007.

41. Schmoller KM, Lanz MC, Kim J, Koivomagi M, Qu Y, Tang C, et al. Whi5 is diluted and protein synthesis does not dramatically increase in pre-Start G1. Molecular biology of the cell. 2022;33(5):lt1. doi:10.1091/mbc.E21-01-0029.

42. Litsios A, Goswami P, Terpstra HM, Coffin C, Vuillemenot LA, Rovetta M, et al. The timing of Start is determined primarily by increased synthesis of the Cln3 activator rather than dilution of the Whi5 inhibitor. Molecular biology of the cell. 2022;33(5):rp2. doi:10.1091/mbc.E21-07-0349.

43. Tollis S. The G1/S repressor WHI5 is expressed at similar levels throughout the cell cycle. BMC Research Notes. 2022;15(1):248. doi:10.1186/s13104-022-06142-9.

Table A: A summary of the parameters used in the simulations if not mentioned in the text. Note that some of the parameters may not be realistic estimations of any specific organisms and our main conclusions are independent of the chosen parameters.

Variables	Meaning	Values
K_n	average transcriptional MM constant	6000 μm^{-3}
K_r	translational MM constant	6000 μm^{-3}
v_n	RNAP elongation speed	36 nt/s
v_r	ribosome elongation speed	12 aa/s
g_n	copy number of the RNAP gene	1
g_r	copy number of the ribosome gene	5
g_i $(i > 2)$	copy number of other genes	1
L_n	number of amino acids of the RNAP	10^3 aa
L_r	number of amino acids of the ribosome	10^4 aa
L	number of amino acids of other proteins	500 aa
ρ	protein mass per cell volume	10^{10} aa/μm^{-3}
a	ratio between cell volume and nuclear volume	20
N	total number of genes	2000
Γ_r	translational initiation rate	10 min^{-1}
τ_m	lifetime of mRNAs	5 min
τ_p	lifetime of degradable proteins	5 min
-	lifetime of nondegradable proteins	10^4 min
T_M	time between gene replication and cell division	60 min
