# Supplementary figures and images for: Implications of differential size-scaling of cell-cycle regulators on cell size homeostasis
Source: PLoS Comput Biol. 2023 Jul 28;19(7):e1011336. doi: 10.1371/journal.pcbi.1011336 (PMC10411824; doi:10.1371/journal.pcbi.1011336)

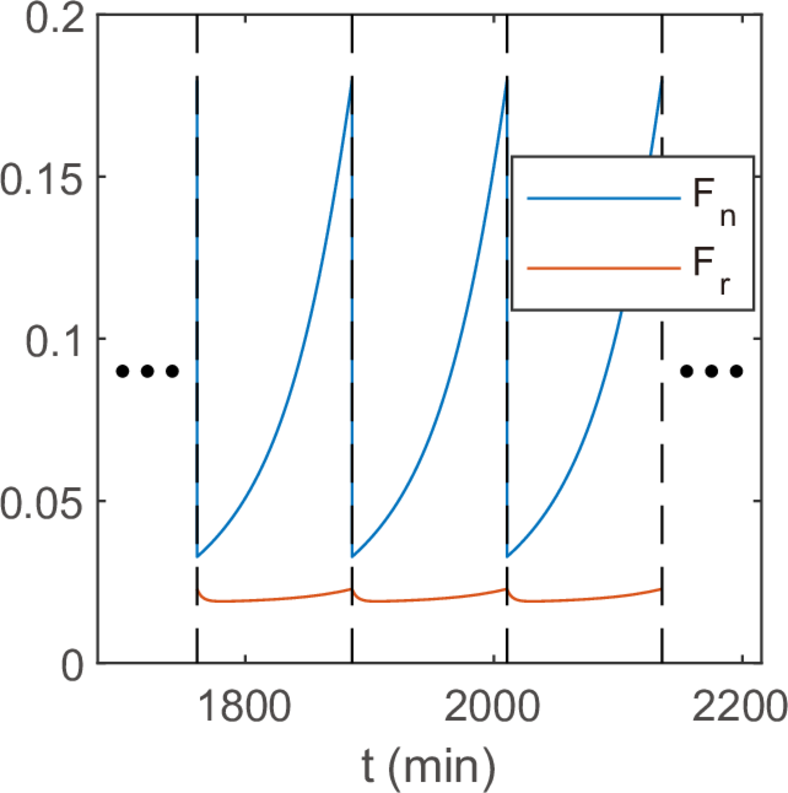

Supplement: S1 Fig — Kn,act = 12000 μm-3, Kn,inh = 4000 μm-3, and θ = 0.8. (TIF) [file pcbi.1011336.s002.tif]

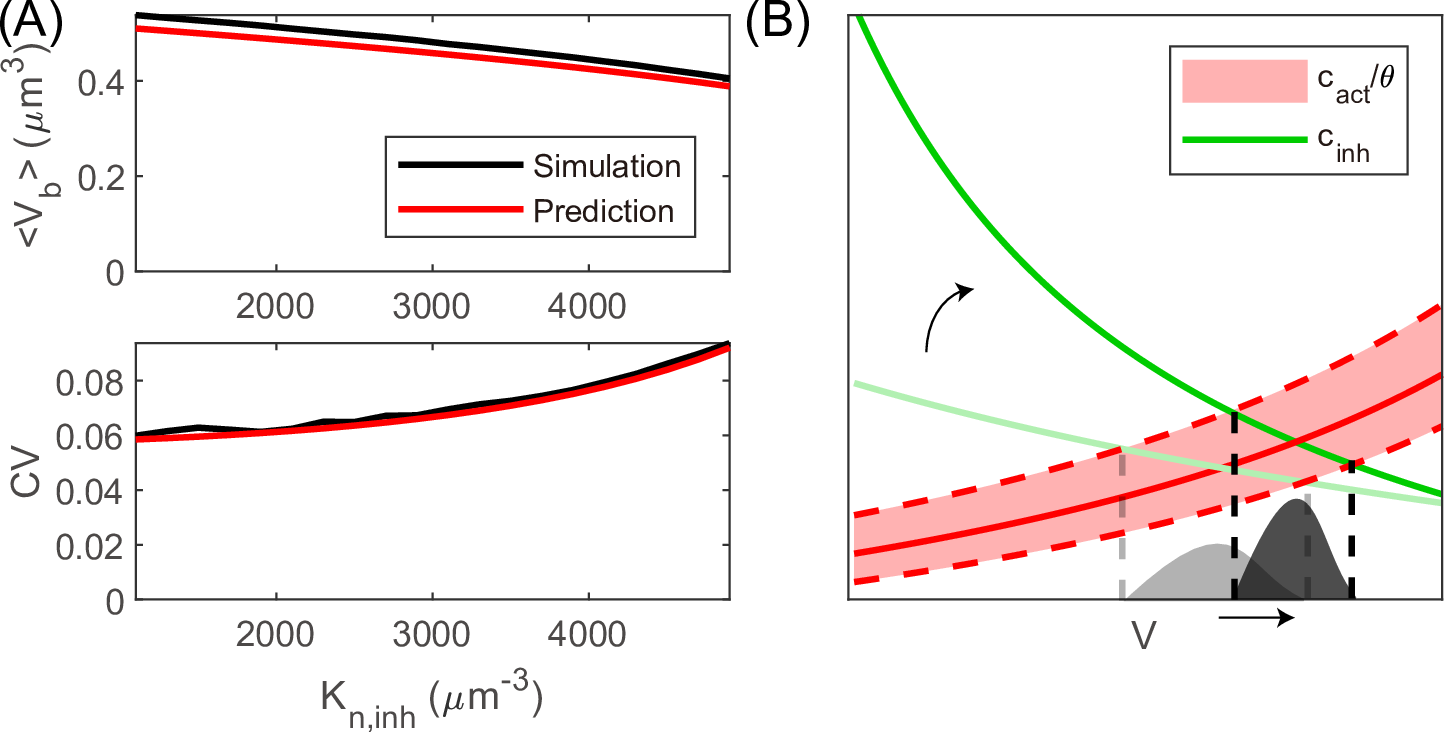

Supplement: S2 Fig — (A) 〈Vb〉 decreases with Kn,inh, and the CV of Vb increases with Kn,inh. Kn,act = 12000 μm-3, θ¯=0.7, Δθ = 0.1. (B) Illustration of the tendency in (A). The intersection of cinh and cact/θ¯ (red solid line) determines 〈Vd〉. The intersections of cinh with cact/(θ¯-Δθ) and cact/(θ¯+Δθ) (red dashed lines) set the range of Vd. If Kn,inh decreases, the number of inhibitor becomes more sublinear as a function of cell size and cinh shifts to the green line with lower transparency. Therefore, 〈Vd〉 increases while the CV of Vd decreases. Since Vb = Vd/2, the same conclusions apply to Vb. (TIF) [file pcbi.1011336.s003.tif]

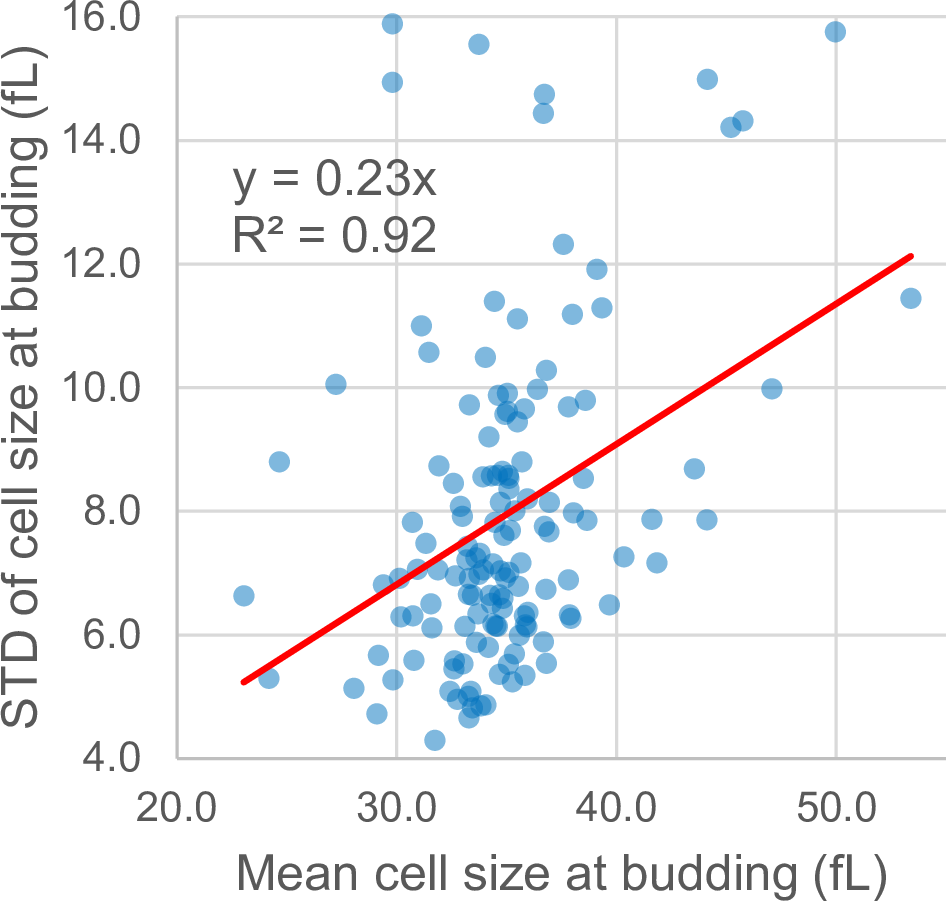

Supplement: S3 Fig — Each point represent a budding yeast mutant with one particular gene belonging to the positive or negative regulator category [52] knocked out, and the red line is the linear fit using the equation y = bx. The R2 is about 0.92, indicating that the STD is nearly proportional to the mean. Thus, after deleting an activator or inhibitor, the mean cell size at budding can change a lot while the CV of cell size at budding remains roughly the same. The data are from Dataset S3 of [52]. (TIF) [file pcbi.1011336.s004.tif]

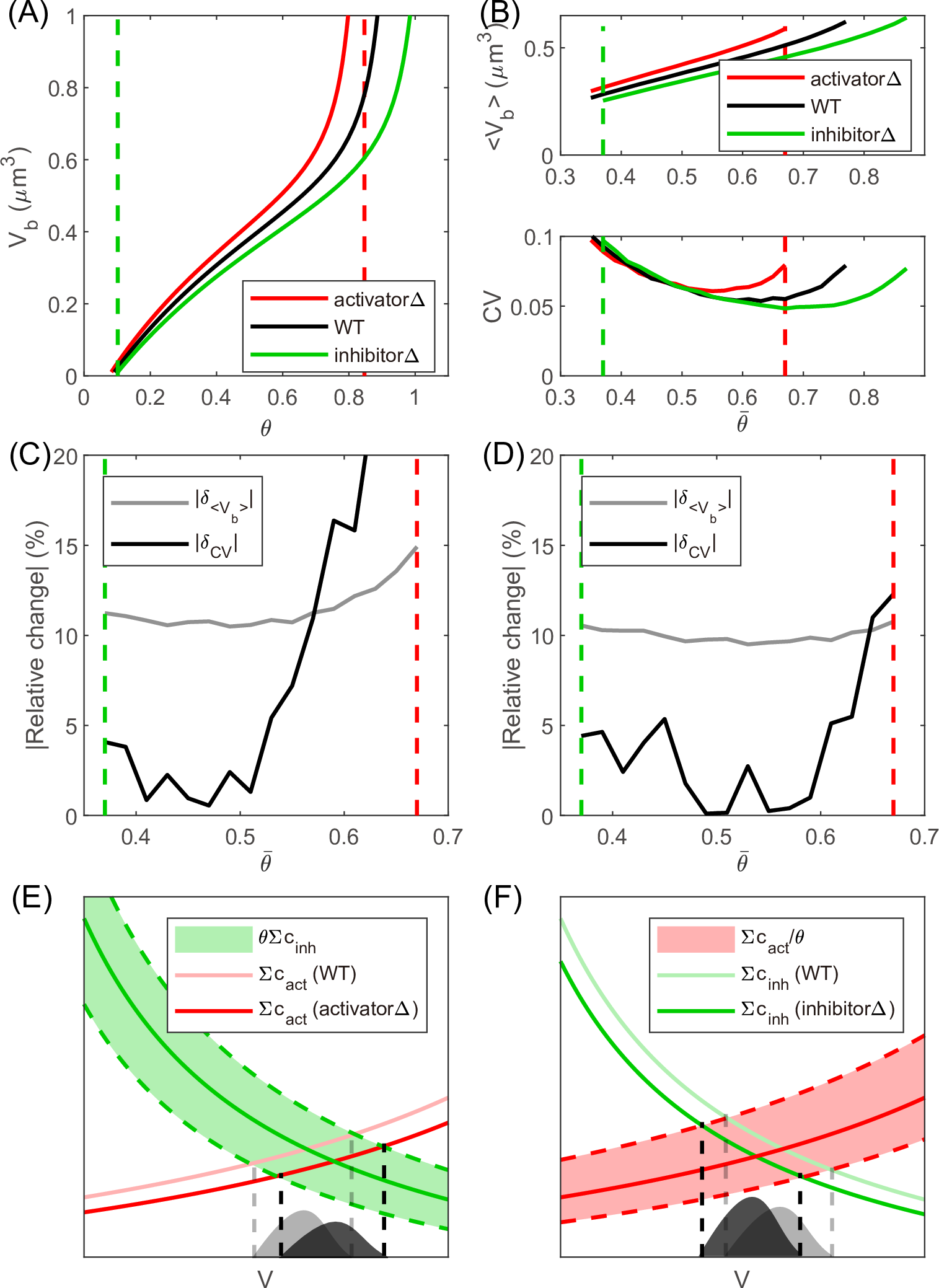

Supplement: S4 Fig — (A) Given a fixed θ, Vb increases after deleting one activator and decreases after deleting one inhibitor. The dashed lines mark the range of θ that simultaneously allows WT, activatorΔ and inhibitorΔ to divide. (B) In the stochastic model, 〈Vb〉 increases after deleting one activator and decreases after deleting one inhibitor. The CV of Vb changes mildly after deleting one regulator. The dashed lines mark the range of θ¯ that simultaneously allows WT, activatorΔ and inhibitorΔ to divide. (C) The absolute value of the relative change δ〈Vb〉≡〈Vb,Δ〉-〈Vb,WT〉〈Vb,WT〉 and δCV≡CVΔ-CVWTCVWT after deleting one activator. (D) |δ〈Vb〉| and |δCV| vs. θ¯ after deleting one inhibitor. The dashed lines in (C-D) have the same meaning as panel (B). There exists a range of viable θ¯ such that the relative change of CV is smaller than that of 〈Vb〉 after regulator deletion. In (B-D), Δθ = 0.1. In (A-D), Kn,act = 12000 μm-3, Kn,inh = 1000 μm-3, gact = ginh = 10. (E) The deletion of one activator shifts the total concentration of all activators (∑cact) downward while the total concentration of all inhibitors (∑cinh) remains the same. This leads to a change in the mean cell size at division 〈Vd〉 while the CV of Vd is almost the same. (F) The deletion of one inhibitor shifts the total concentration of all inhibitors (∑cinh) downward while the total concentration of all activators (∑cact) remains the same. This leads to a change in 〈Vd〉 while the CV of Vd is almost the same. (TIF) [file pcbi.1011336.s005.tif]

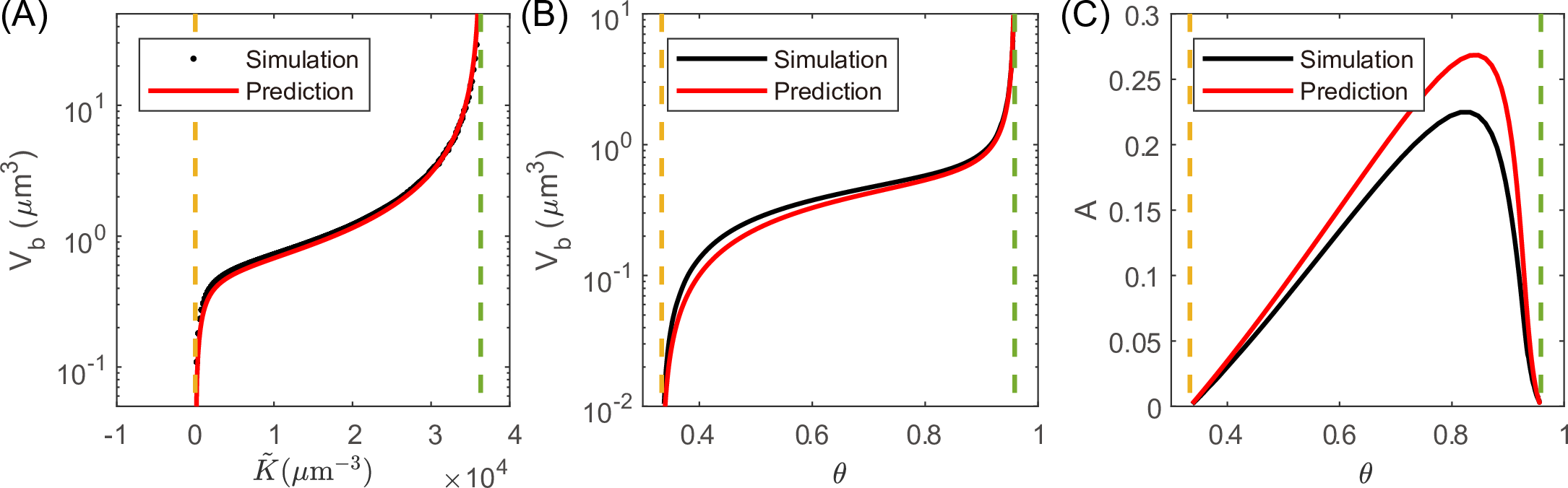

Supplement: S5 Fig — We simulate a model in which the transcription MM constants are heterogeneous among genes, and compare the simulations with theoretical predictions. (A) Vb vs. K˜. θ = 0.8. (B) Vb vs. θ. (C) A vs. θ. In (B-C), Kn,act = 12000 μm-3, Kn,inh = 4000 μm-3. In all panels, the dashed lines mark the two predicted critical threshold values. Kn,i follows a lognormal distribution with the mean Kn = 6000 μm-3 and the CV equal to 0.5. (TIF) [file pcbi.1011336.s006.tif]

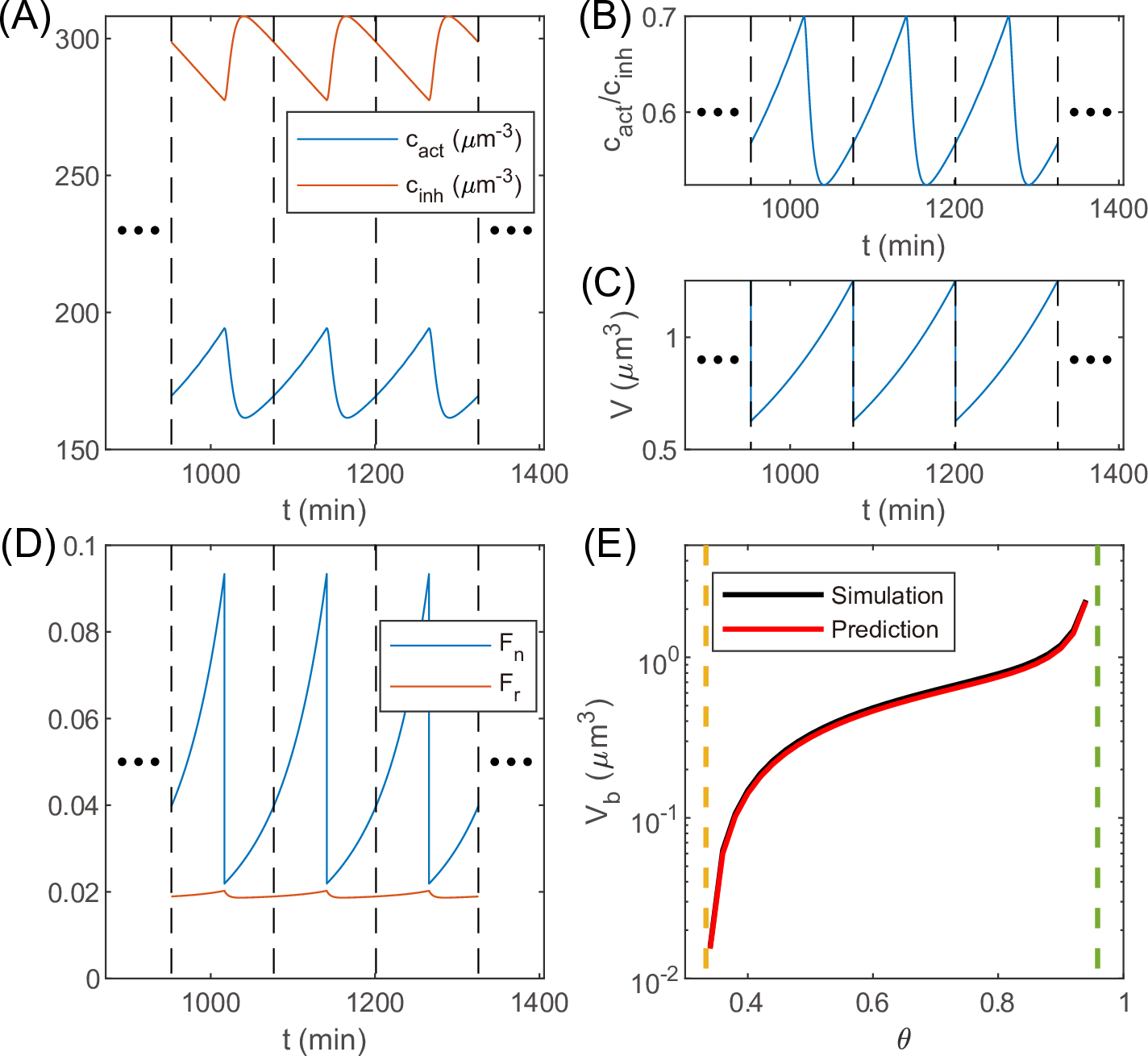

Supplement: S6 Fig — (A) The concentration of the activator cact and inhibitor cinh, (B) cact/cinh and (C) the cell volume V change periodically. (D) The free RNAP fraction Fn drops abruptly at gene replication while the free ribosome fraction Fr does not. In (A-D), the dashed lines mark cell division, and θ = 0.7. (E) Simulations and theoretical predictions of Vb vs. θ. In this figure, Kn,act = 12000 μm-3, Kn,inh = 4000 μm-3, TM = 60 min. (TIF) [file pcbi.1011336.s007.tif]

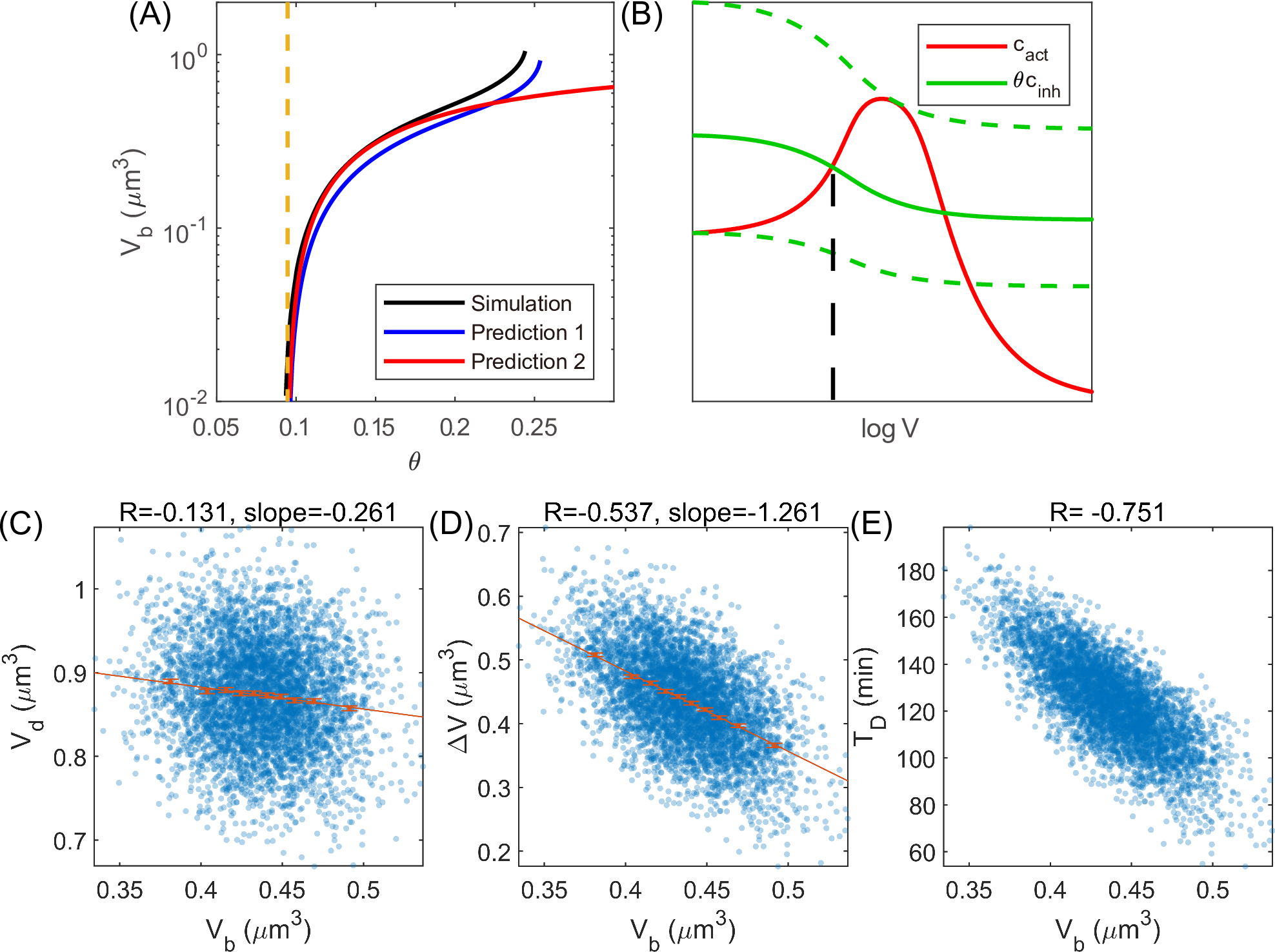

Supplement: S7 Fig — (A) Simulations and theoretical predictions of Vb vs. θ in the deterministic model. The dashed line marks the predicted critical point θ1. We do not plot the predicted critical point θ2 because it does not have an analytical expression. (B) Illustration of two critical division thresholds. Note that cact and θcinh may have two intersections. Only the intersection on the left determines Vd, because as the cell volume increases, cact/cinh must rise to θ. Beyond two critical points (dashed lines), there is no intersection on the left. (C) Vd vs. Vb in the stochastic model. (D) ΔV vs. Vb. (E) TD vs. Vb. In (C-E), we simulate the stochastic model and each point represents one cell cycle. The Pearson correlation coefficient R and slope of the linear regression on binned data are shown in the title. Each bin includes an equal number of data points. Error bars: mean±SEM. θ¯=0.18, Δθ = 0.018. In (A, C-E), Kn,act = 12000 μm-3, Kn,inh = 4000 μm-3, α = 10. (TIF) [file pcbi.1011336.s008.tif]

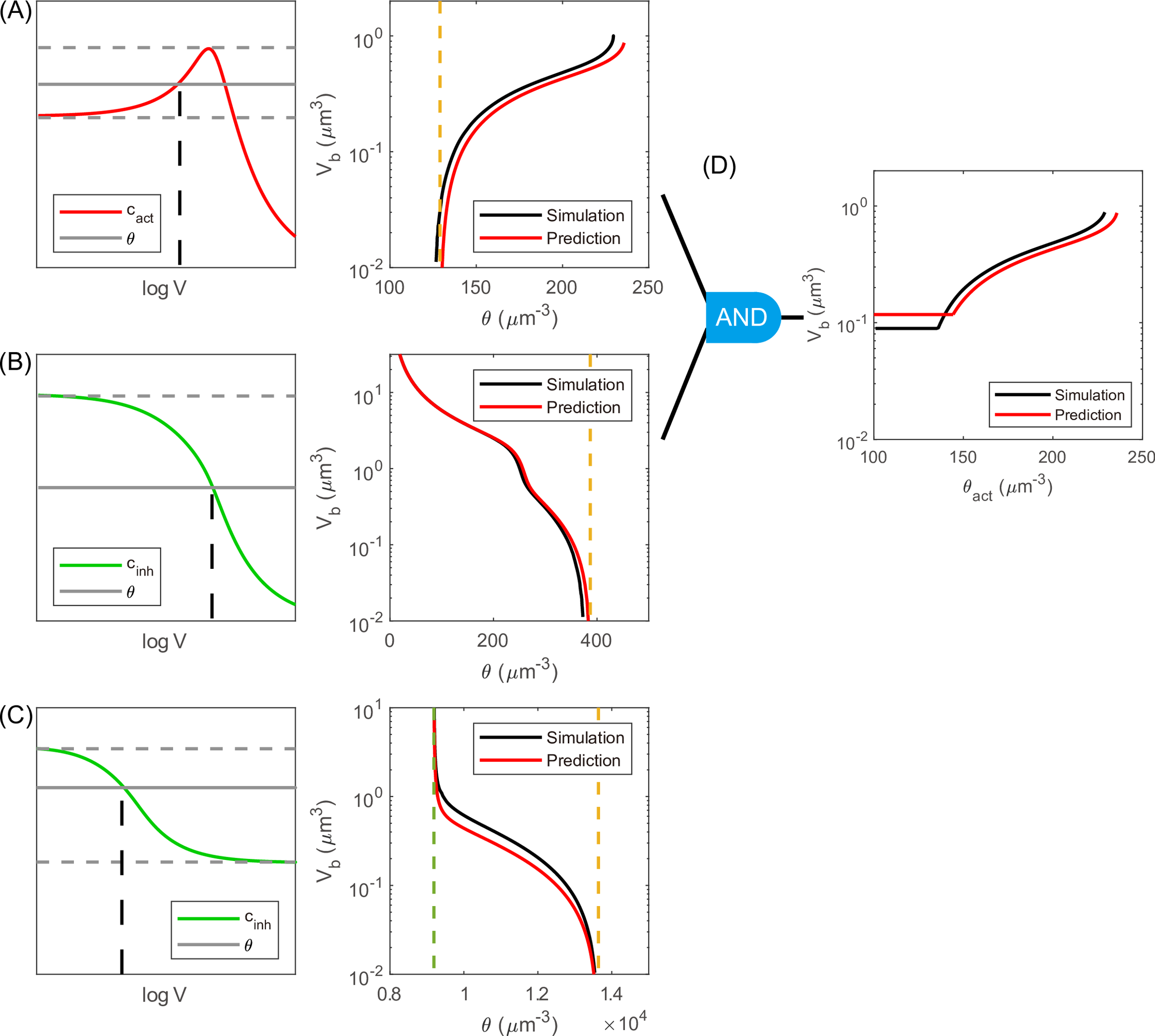

Supplement: S8 Fig — (A) In this modified model, the cell divides once the activator concentration cact increases to the threshold θ, and the activator is degradable. (B-C) The cell divides once the inhibitor concentration cinh decreases to the threshold θ. The inhibitor is degradable in (B) and nondegradable in (C). In each panel, on the left is cact (cinh) vs. V, illustrating the origins of the critical points. The intersection of cact (cinh) and θ (the solid gray line) determines the cell size at division. Note that cact and θ may have two intersections. Only the intersection on the left determines Vd, because as the cell volume increases, cact must rise to θ. There is no intersection beyond the critical threshold values (gray dashed lines). On the right is the cell size at birth Vb vs. θ. The dashed lines mark the predicted critical threshold values. (D) The cell integrates the information of one activator and one inhibitor using the AND logic. It divides once both cact and cinh reach their own thresholds. Here we show Vb vs. the activator’s threshold θact given a fixed threshold θinh = 350 μm-3 for the inhibitor. In this figure, Kn,act = 12000 μm-3, Kn,inh = 4000 μm-3. (TIF) [file pcbi.1011336.s009.tif]

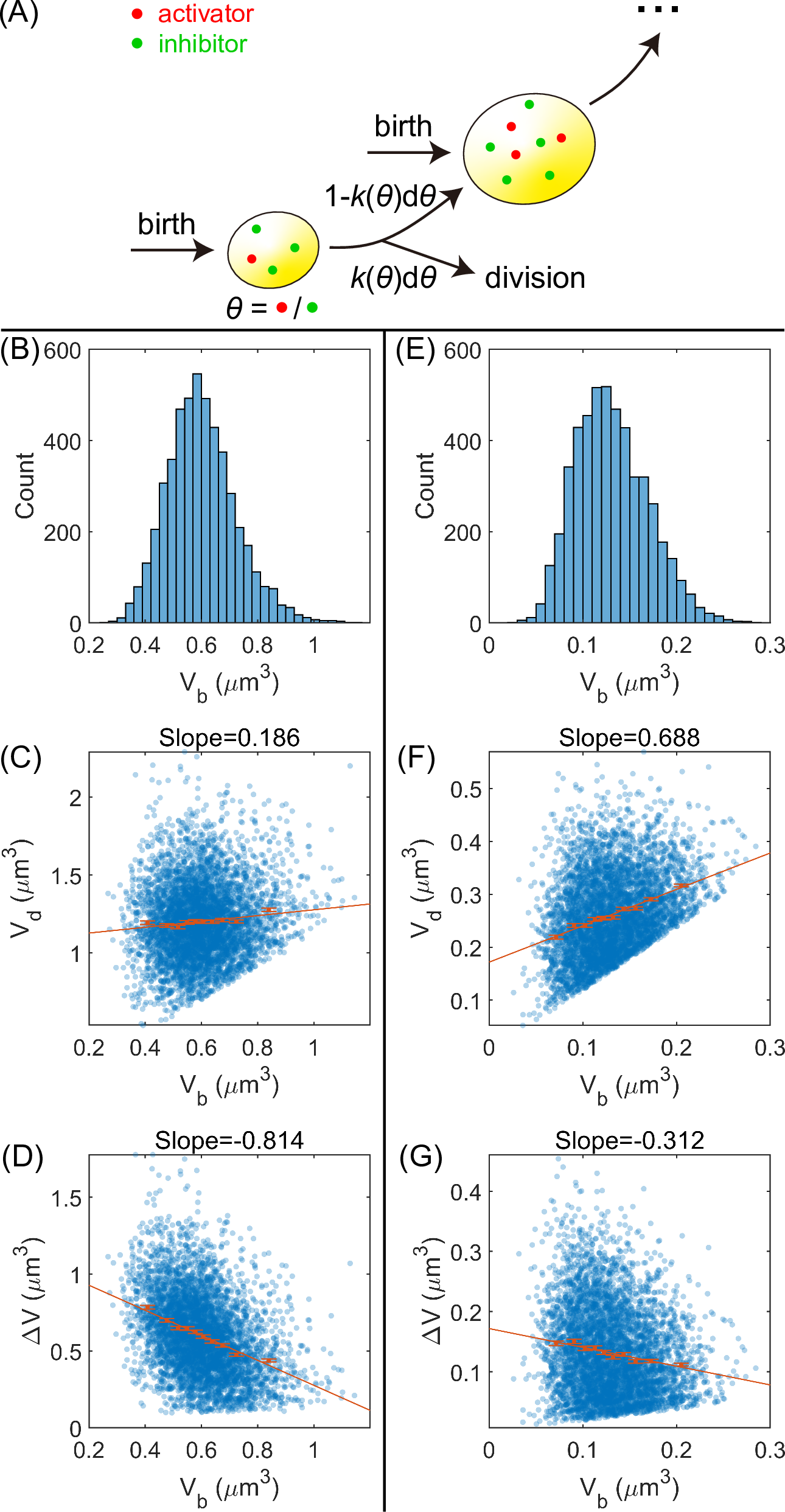

Supplement: S9 Fig — (A) We assume that the probability for a cell with the activator-to-inhibitor ratio θ in the infinitesimal interval [θ,θ+dθ) to divide is k(θ)dθ. The cell born with a smaller size is more likely to divide at a smaller size because it has a finite probability to divide before it reaches the birth size of the larger cell. (B-G) Simulations of Vb and Vd. We simulate 5000 generations and trace one of the two daughter cells after division. For each generation, we first let the cell grow for a minimal time (20 min), which has minor effect on the slope of Vd vs. Vb. We then use inverse transform sampling to generate the random variable θ, which is the activator-to-inhibitor ratio at cell division and has a probability density function p(θ) (see S1 Appendix for more details). In inverse transform sampling, we first take a random number x from a uniform distribution between 0 and 1. θ is then solved from F(θ) = x, where F(θ)=1-e-∫θbθk(θ′)dθ′ is the cumulative distribution function of θ. (B-D) C1 = 1, C2 = 2. The slope is similar to the imperfect sizer in [40, 43–45]. (E-G) C1 = 200, C2 = 1. The slope is similar to the near-adder in [46]. In (C-D,F-G), each bin includes an equal number of data points. Error bars: mean±SEM. In this figure, Kn,act = 12000 μm-3, Kn,inh = 4000 μm-3. (TIF) [file pcbi.1011336.s010.tif]

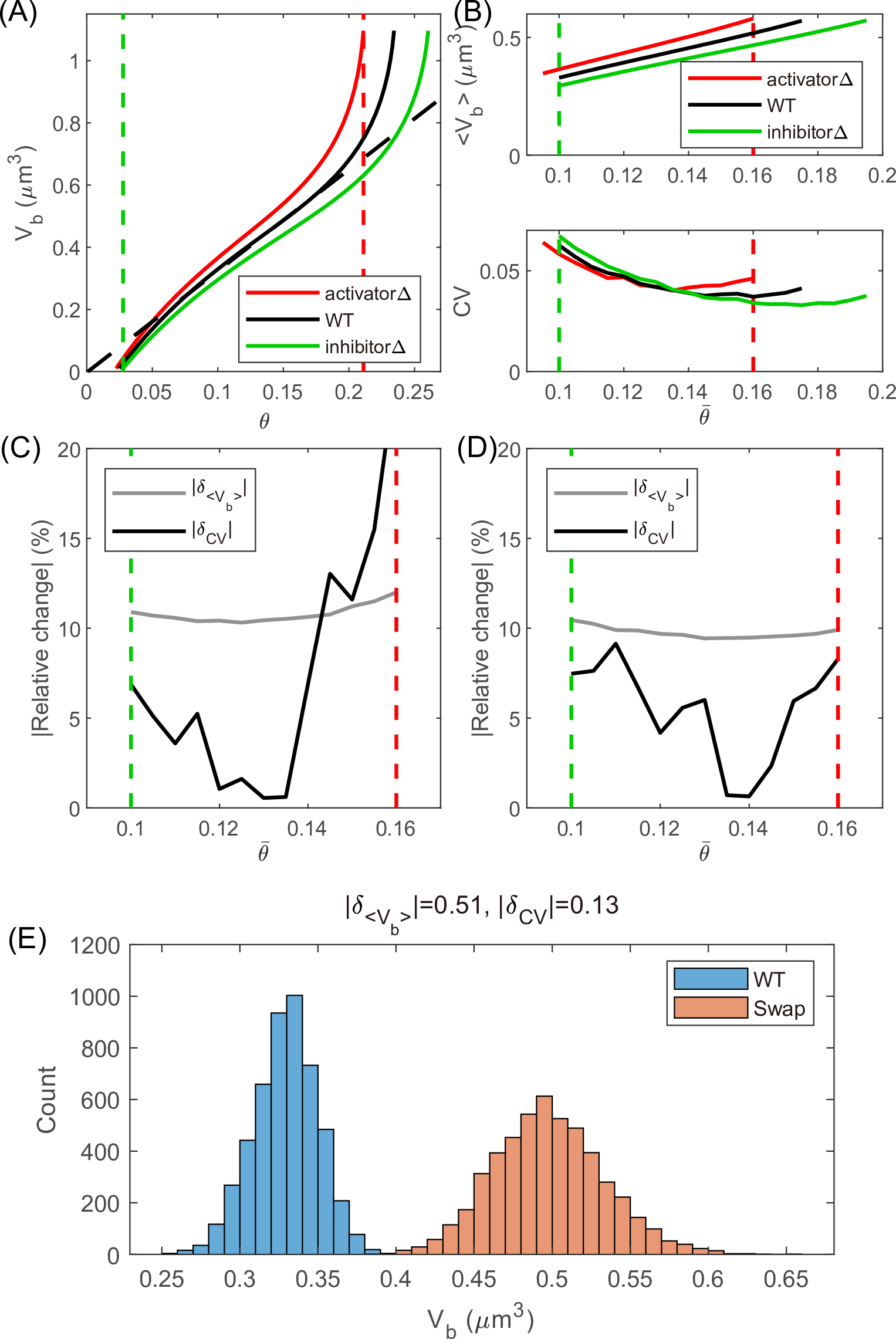

Supplement: S10 Fig — (A) Given a fixed θ, Vb increases after deleting one activator and decreases after deleting one inhibitor. The green and red dashed lines mark the range of θ that simultaneously allows WT, activatorΔ and inhibitorΔ to divide. The black dashed line marks the linear relationship between Vb and θ in a wide range. gact = ginh = 10. (B) In the stochastic model, 〈Vb〉 increases after deleting one activator and decreases after deleting one inhibitor. The CV of Vb changes mildly after deleting one regulator. (C) |δ〈Vb〉| and |δCV| vs. θ¯ after deleting one activator. (D) |δ〈Vb〉| and |δCV| vs. θ¯ after deleting one inhibitor. In (B-D), the dashed lines mark the range of θ¯ that simultaneously allows WT, activatorΔ and inhibitorΔ to divide. (E) The distribution of cell size at birth Vb for WT and the mutant with the promoters of one activator and one inhibitor swapped. The average cell size and CV increase compared with the wild type, consistent with experiments [12]. θ¯=0.1. In this figure, Kn,act = 12000 μm-3, Kn,inh = 1000 μm-3, Δθ = 0.009, α = 10. (TIF) [file pcbi.1011336.s011.tif]

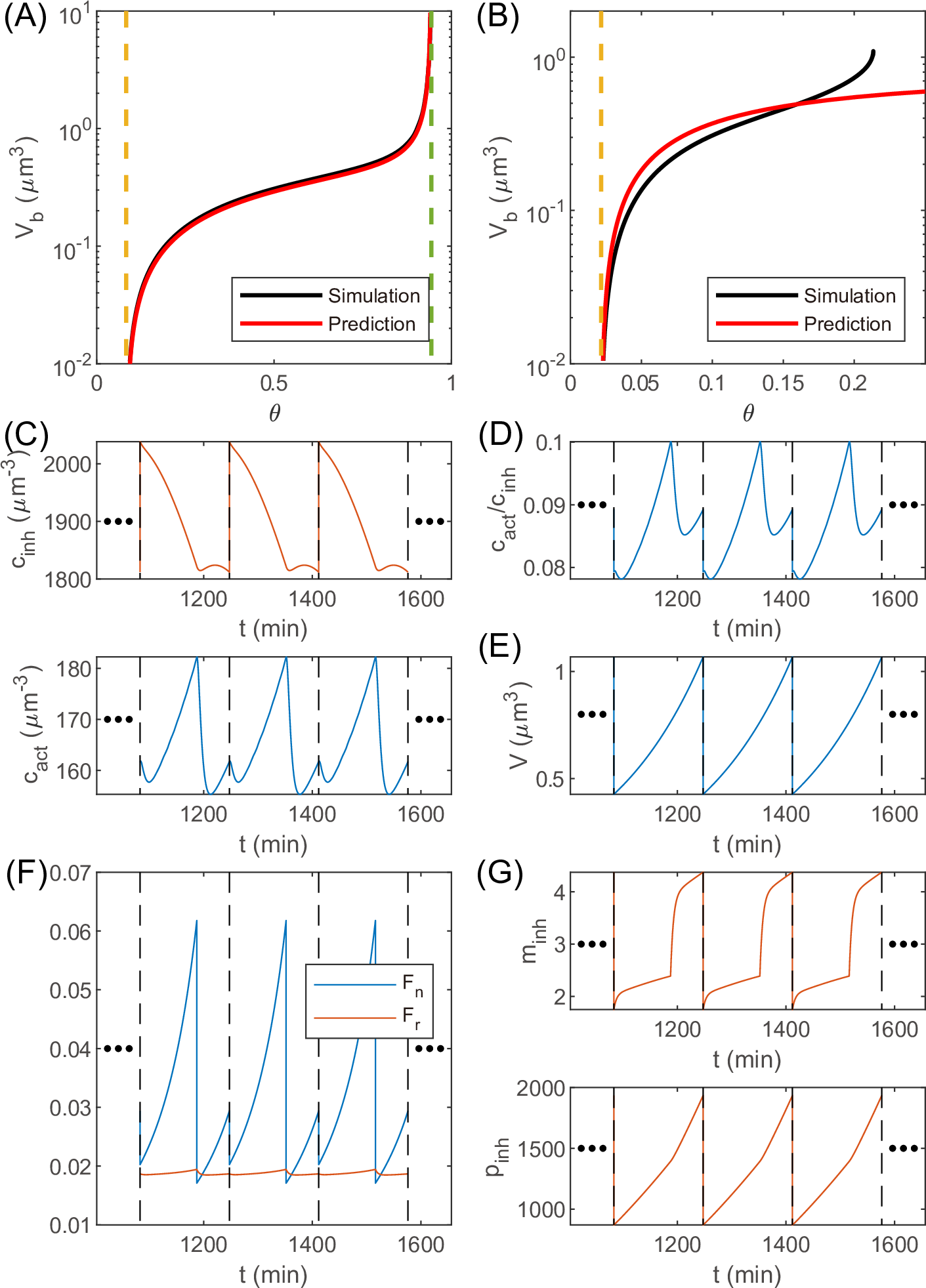

Supplement: S11 Fig — The cell divides asymmetrically with γ = 0.4. We track the daughter cell and simulate a single lineage to the steady state (see details in S1 Appendix). (A) Simulations and theoretical predictions of the cell size at birth Vb vs. the division threshold θ for the case of degradable regulators. (B) Simulations and theoretical predictions of Vb vs. θ for the case of degradable activator and nondegradable inhibitor. (C-G) The cell replicates its genes when the activator-to-inhibitor ratio rises to a threshold and then divides after a constant time TM = 60 min. The inhibitor is nondegradable. (C) Simulations of cact and cinh. The dashed lines mark cell division. (D) cact/cinh. (E) V. (F) Fn and Fr. (G) minh and pinh. In (B-G), the inhibitor is distributed between the mother and daughter cells at division as Whi5 in budding yeast, with η = 0.45 [9]. α = 10. In this figure, Kn,act = 12000 μm-3, Kn,inh = 1000 μm-3. (TIF) [file pcbi.1011336.s012.tif]

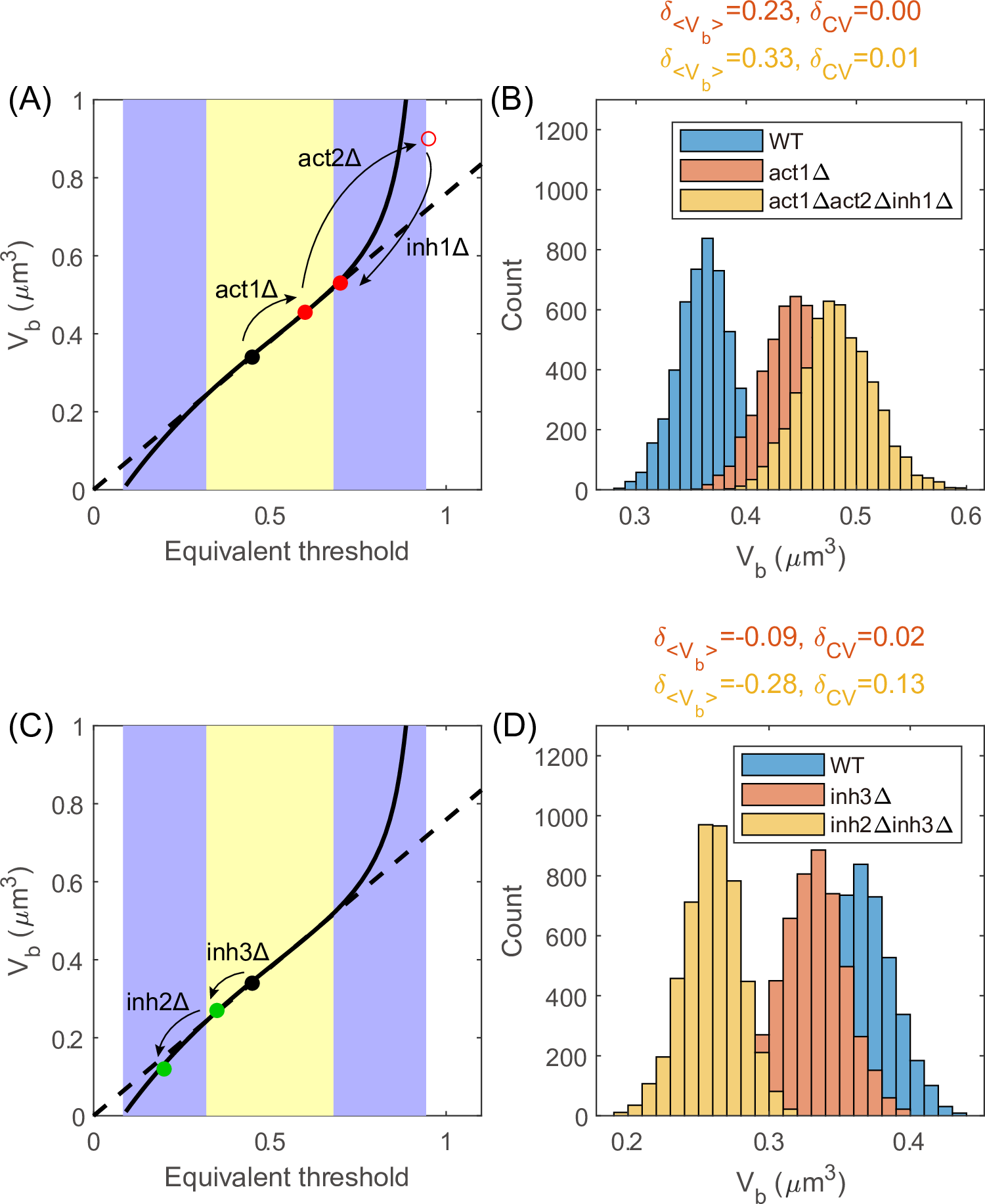

Supplement: S12 Fig — (A) In the model of multiple regulators, the deletion of activators corresponds to an increase in the equivalent division threshold. If the threshold change is small, e.g., act1Δ, the cell size at birth Vb is still proportional to the equivalent threshold (the yellow region) so that the CV of Vb remains constant. If the threshold change is significant, e.g., act1Δ act2Δ, the equivalent threshold is beyond the critical division threshold (the white region), and the cell becomes inviable (the red hollow circle). However, additional deletion of inh1 rescues the cell. (B) The distributions of Vb for WT, act1Δ and act1Δ act2Δ inh1Δ. (C) The deletion of inhibitors is equivalent to a decrease in the division threshold. If the threshold change is small, e.g., inh3Δ, Vb is still proportional to the equivalent threshold so that the CV of Vb remains constant. If the threshold change is significant, e.g., inh2Δ inh3Δ, Vb becomes a nonlinear function of the equivalent threshold (the purple region), and the CV increases. (D) The distributions of Vb for WT, inh3Δ, and inh2Δ inh3Δ. In (B, D), the mutants’ relative changes in average cell size and CV compared with WT are shown at the top. In simulations, we introduce different weights χact,i and χinh,i for different regulators. The cell divides once ∑i χact,icact,i/∑ χinh,icinh,i = θ so that the change of average cell size can be different after deleting different activators or inhibitors. The Kn,act of 10 activators is an arithmetic sequence from 9000 to 15000 μm-3. The Kn,inh of 10 inhibitors is an arithmetic sequence from 750 to 1250 μm-3. Meanwhile, we also introduce heterogeneous Kn,i for other genes except the cell-cycle regulators, which follows a lognormal distribution with the mean Kn = 6000 μm-3 and the CV equal to 0.5. Our conclusion regarding the CV of cell size after gene deletions remains valid in the presence of heterogeneous Kn,i. χact,1 = χact,2 = 2, χact,3 = … = χact,6 = 1, χact,7 = … = χact,10 = 0.5. χinh,1 = χinh [file pcbi.1011336.s013.tif]
